# Transcolonic endoscopic appendectomy using a novel integrated snare-knife device: a case report

**DOI:** 10.1055/a-2752-9749

**Published:** 2026-01-08

**Authors:** Tingxuan Huang, Fenglin Chen, Xin-Yang Liu, Xiaoxiong Guo, Ping-Hong Zhou

**Affiliations:** 1 117890 Department of Gastroenterology, Fujian Medical University Union Hospital, Fuzhou, China; 2 Fujian Clinical Research Center for Digestive System Tumors and Upper Gastrointestinal Diseases, Fuzhou, China; 3 92323 Endoscopy Center and Endoscopy Research Institute, Zhongshan Hospital, Fudan University, Shanghai, China

**Fig. 1:**
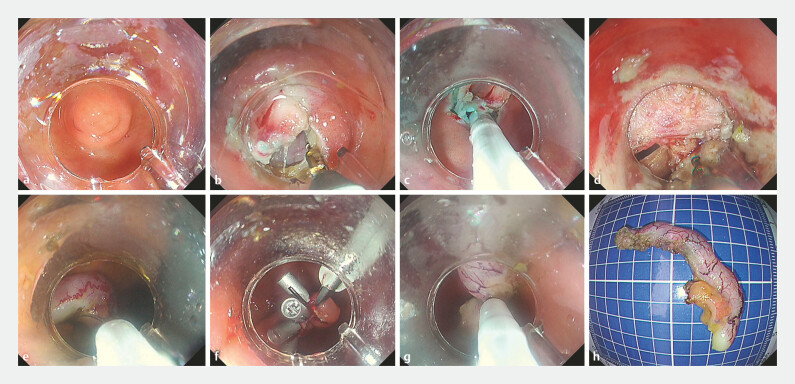
Transcolonic endoscopic appendectomy using the snare probe throughout the procedure. **a** Protruding lesions adjacent to the appendix. **b** Mucosal incision. **c** Submucosal injection. **d** Coagulation for hemostasis. **e** Conversion to the snare for appendiceal resection. **f** Application of the traction clip for wound closure. **g** Appendix removed by snare mode. **h** The resected specimen.

A 57-year-old woman presented with an incidental 5 mm protrusion at the appendiceal orifice, identified during a routine examination. The asymptomatic patient had no notable medical history. Under non-intubated anesthesia with the patient positioned in the left lateral decubitus position, the procedure was initiated with a submucosal injection utilizing the integrated needle mode. Furthermore, a circumferential incision was created followed by full-thickness dissection with intermittent electrocoagulation. Meanwhile, the minimal purulent material was encountered during peritoneal entry. The appendiceal mesentery was systematically divided utilizing the knife function, followed by complete resection of the appendix and specimen retrieval with the integrated snare. Post-resection, the enterotomy was closed utilizing a glove-derived rubber band traction system combined with endoscopic suturing. Additionally, a drainage catheter was deployed under direct visualization ( Fig. 1 ). Within 30 minutes, appendiceal resection was completed and the total procedural duration was only 60 minutes ( Video 1 ).

Transcolonic endoscopic appendectomy using a novel integrated snare-knife device.Video 1

**Fig. 2:**
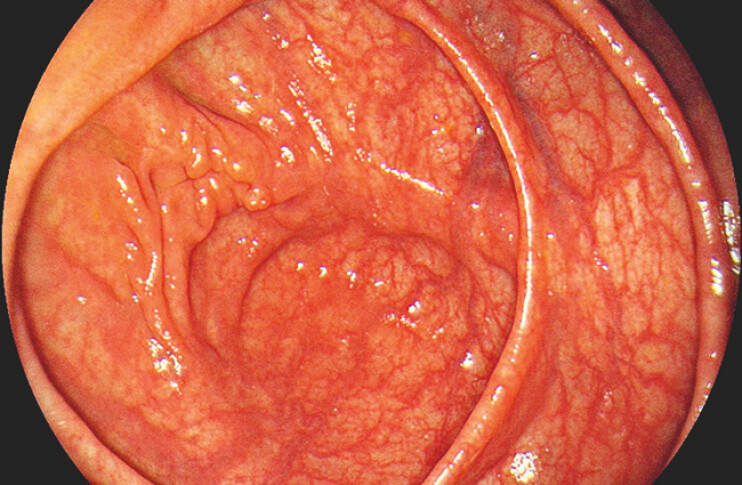
Colonoscopy images at a 4-month postoperative follow-up.

Histopathological analysis confirmed chronic inflammation of the appendix with associated abscess formation. The patient experienced no postoperative discomfort. A follow-up colonoscopy performed 4 months later demonstrated good healing of the surgical incision ( Fig. 2 ).

This represents the first documented utilization of a multifunctional snare probe combining the electrosurgical knife, snare, and injection needle capabilities in NOTES appendectomy 1 2 3 .

Endoscopy_UCTN_Code_TTT_1AT_2AZ
